# One Welfare in Practice: The Role of the Veterinarian

**DOI:** 10.1017/awf.2023.13

**Published:** 2023-02-27

**Authors:** Claire Richardson

**Affiliations:** UFAW, Wheathampstead, UK

*One Welfare in Practice: The Role of the Veterinarian* forms part of a series of books published by CRC One Health One Welfare which explore interconnections between human well-being, animal health and welfare and the environment. It is edited by Dr Tanya Stephens who is a small animal practitioner, wildlife researcher and fellow of the Royal College of Veterinary Surgeons living and working in Australia. The book consists of 14 chapters each offering a slightly different perspective on the key role that veterinarians can play in promoting one welfare. Chapters fall into three broad categories including veterinary roles in: (i) environmental issues; (ii) humane applications of population control measures; and (iii) promoting animal welfare. Most of the authors are from universities or research institutions in Australia or New Zealand but the focus is on One Welfare globally.

The first chapters relate to the role of veterinarians in environmental issues. This was the area that I was least familiar with and one that did not feature in the veterinarian curriculum when I attended vet school twenty years ago. A helpful starting point in this field was the overview of the Vet Sustain group (https://vetsustain.org/) which aims to promote the role of veterinarians in aligning animal, human and environmental well-being. The chapter included a summary of their six sustainability goals as well as several case studies, including the ‘3Rs goals of antibiotic stewardship’ plus key links and further reading. Other chapters include discussions of the partnership between one health and welfare, consideration of climate change as an animal welfare problem as well as animal welfare aspects of land clearing.

The next group of chapters relate to humane applications of population control measures included tuberculosis control in New Zealand, rabies control in Indonesia as well as vertebrate ‘pest’ control which is an area of particular interest for UFAW (https://www.ufaw.org.uk/rodent-welfare/rodent-welfare). The focus of the chapter on kangaroos in the vertebrate ‘pest’ animals chapter was specific to Australia, but the general message of a holistic approach involving a range of different communities often with different aims, attitudes and perceptions apply generally to the complexity of ‘pest’ animal management in any species and geographical area.

The final group of chapters relate to the veterinarian’s role in promoting animal welfare including chapters on laboratory animals, fish, cattle and working animals as well as transport and nutritional security. Of particular interest to me was the chapter on laboratory animals. Dr Alexandra Whittaker of the University of Adelaide outlines the roles that lab animal veterinarians can play in promoting one health and welfare and provides a general introduction to this role of a laboratory animal veterinarian as well as ethical issues associated with this work. The chapter ends with a case study on research into novel methods of vertebrate pest control which links nicely with other chapters in the book.

Overall, I would recommend this book to those with an interest in One Welfare. Although the focus is on the role of the veterinarian, it is clear throughout the book that vets cannot work in isolation and that solutions can only be found by communities and professions coming together and working towards shared goals. This book is likely to be of interest to all of us with an interest in animal, human and environmental health and well-being. It is likely to be of particular interest to veterinary students and recent graduates as it highlights the breadth of potentially diverse roles that veterinarians can have in promoting one health and welfare.

